# Intravital imaging of cardiac tissue utilizing tissue-stabilized heart window chamber in live animal model

**DOI:** 10.1093/ehjimp/qyae062

**Published:** 2024-07-01

**Authors:** Soyeon Ahn, Jung-yeon Yoon, Pilhan Kim

**Affiliations:** R&D Center, IVIM Technology, 17 Techno 4-ro, Yuseong-gu, Daejeon, 34013, Republic of Korea; R&D Center, IVIM Technology, 17 Techno 4-ro, Yuseong-gu, Daejeon, 34013, Republic of Korea; R&D Center, IVIM Technology, 17 Techno 4-ro, Yuseong-gu, Daejeon, 34013, Republic of Korea; Graduate School of Medical Science and Engineering, Korea Advanced Institute of Science and Technology (KAIST), 291 Daehak-ro, Yuseong-gu, Daejeon, 34141, Republic of Korea

**Keywords:** intravital imaging, cardiovascular imaging, live heart imaging, *in vivo* cardiac imaging

## Abstract

**Aims:**

To develop and validate an optimized intravital heart microimaging protocol using a suction-based tissue motion-stabilizing cardiac imaging window to facilitate real-time observation of dynamic cellular behaviours within cardiac tissue in live mouse models.

**Methods and results:**

Intravital heart imaging was conducted using dual-mode confocal and two-photon microscopy. Mice were anesthetized, intubated, and maintained at a stable body temperature during the procedure. LysM-eGFP transgenic mice were utilized to visualize immune cell dynamics with vascular labelling by intravenous injection of anti-CD31 antibody and DiD-labelled red blood cells (RBCs). A heart imaging window chamber with a vacuum-based tissue motion stabilizer with 890–920 mbar was applied following a chest incision to expose the cardiac tissue. The suction-based heart imaging window chamber system and artificial intelligence-based motion compensation function significantly reduced motion artefacts and facilitated real-time *in vivo* cell analysis of immune cell and RBC trafficking, revealing a mean neutrophil movement velocity of 1.66 mm/s, which was slower compared to the RBC flow velocity of 9.22 mm/s. Intravital two-photon microscopic heart imaging enabled label-free second harmonic generation imaging of cardiac muscle structures with 820–840 nm excitation wavelength, revealing detailed biodistributions and structural variations in sarcomeres and fibrillar organization in the heart.

**Conclusion:**

The optimized intravital heart imaging protocol successfully demonstrates its capability to provide high-resolution, real-time visualization of dynamic cellular activities within live cardiac tissue.

## Introduction

Cardiovascular disease is one of the most fatal diseases in the current era, with high mortality. Extensive preclinical and clinical studies have aimed to understand underlying cellular and molecular mechanisms in cardiovascular disease.^1–7^ There are several research imaging modalities for diagnosis and monitoring of live cardiac tissue in clinical and preclinical studies including ultrasound,^8–10^ magnetic resonance imaging (MRI),^11–16^ computed tomography (CT),^17–19^ and positron emission tomography (PET).^20–22^ However, despite the technological advances, intravital heart imaging technology has predominantly relied on macroscopic techniques, providing primarily structural and functional information while lacking cellular-level dynamics. *In vivo* microscopy in the heart enables the observation of cellular dynamics and physiological interactions under various conditions, facilitating the assessment of immune cell trafficking and vascular integrity alterations. To accomplish this, it is essential to implement practical measures to address motion artefacts resulting from respiratory and cardiac movement, ensuring optimal tissue stabilization. Furthermore, utilizing algorithmic frameworks conducive to real-time acquisition is crucial for obtaining high-fidelity, high-resolution image data sets. Various imaging strategies have been developed, including passive stabilizer with tissue adhesive,^23,24^ suction-based tissue stabilizer,^25^ and microendoscopy integrated with suction probe^26^; however, most of which may cause tissue impairment and physiological alterations and have limited imaging field of view.

Here, we introduce the optimized *in vivo* cellular-level imaging method through heart-specific tissue-stabilizing imaging window chambers for live heart visualization in real time by ultra-fast confocal and two-photon intravital microscopy (IVM) imaging platform. The heart imaging window chamber significantly reduced the motion artefacts generated from the cardiac contraction, minimizing physiological perturbations and allowing for a stable real-time cellular analysis of immune cell trafficking and red blood cell (RBC) flow over the wide area of the heart. Together with artificial intelligence (AI)-based motion compensation function, the real-time acquisition of high-quality *in vivo* imaging data of live heart tissue is fully available in living objects. Moreover, label-free cardiac muscle can be visualized in second harmonic generation (SHG) signals in two-photon IVM with 820–840 nm wavelength excitation settings. In this study, we present an optimized intravital heart imaging method using the cardiac tissue-stabilized imaging window chamber for IVM and imaging applications to visualize cellular dynamics of immune cells, RBCs, and label-free SHG cardiac muscle fibres.

## Methods

### Mice

All mouse experiments were performed according to the ARRIVE (Animal Research: Reporting In Vivo Experiments) guidelines with the protocol approved by the Animal Care and Use Committee of IVIM Technology (IACUC approval no. 2019-06.1). The animal surgeries were carried out under complete anaesthesia status, and every effort was performed to minimize the suffering of a live mouse model. LysM-eGFP genetically engineered transgenic mice expressing GFP in neutrophils and monocytes in cardiac tissue were used for *in vivo* visualization of immune cell dynamics in this research.

### IVM

All-in-one real-time IVM system with dual-mode confocal and two-photon microscopy (IVM-CM, IVIM Technology) was utilized for *in vivo* visualization of heart tissue in live animal models. For confocal microscopy, simultaneous four-colour fluorescent imaging of DAPI, GFP, RFP, and Cy5 is enabled with continuous laser excitation modules (405, 488, 561, and 640 nm). In multiphoton microscopy imaging mode, a tuneable two-photon laser source of 690–100 nm in wavelength was illuminated upon the desired fluorescence options. Label-free SHG signal expressed from cardiac muscles was visualized on 820–840 nm excitation wavelength of two-photon laser. IVM was operated by the image-acquiring software of IVIM Technology, IVM-Engine, and the result image data were processed in 2D–4D by post-processing software, IVM-Studio. AI-based automatic motion compensation functions proceeded with frame-by-frame compensation in reference of selected channel of unmoving targets in the region of interest (ROI). It is dedicated to IVM-Engine software, enabling real-time acquisition of stabilized image sequences of highly moving organs, including the heart and lung, in live animal models.

### Intravital heart imaging using cardiac tissue window chamber

Mouse surgeries for heart imaging window chamber application were performed under anaesthesia with Zoletil (30 mg/kg) and xylazine (10 mg/kg) cocktail mixture. Mouse was intubated through the trachea and connected with a ventilator (MouseVent, Kent Scientific) with 24∼30 mmHg of inspiratory pressure. Mouse body core temperature was stably maintained at 36.5°C by *in vivo* body temperature monitoring and feedback control unit (4CH body and tissue temperature control system, IVIM Technology). For cardiac muscle exposure, mouse chest skin and pectoralis muscle were incised and thoracotomy was proceeded with dissection of the ribs positioned near heart tissue. After the rib incision in a circular shape to expose cardiac tissue, the heart imaging window chamber modified from the previous pulmonary window systems^27–29^ was adjusted to the surface of cardiac tissue covered with an 8 mm round coverslip. A negative vacuum pressure of 890–920 mbar was applied to the imaging chamber space of the heart window through a tubing connected to the tissue motion-stabilized system (IVM-TMS, IVIM Technology) to temporarily stabilize the cardiac tissue during *in vivo* imaging.

To visualize the cardiac vasculature, anti-CD31 antibody conjugated with FSD647 (999-991-0004, In Vivo Labeling Kits, IVIM Technology) was intravenously injected through the tail vein catheter at 2 h previous intravital imaging. Retro-orbitally collected RBCs were labelled with DiD cell membrane dye (999-991-0045, In Vivo Labeling Kits, IVIM Technology), and 40–50 million DiD-labelled RBCs were adoptively transferred to the imaging model via systemic vascular infusion.

## Results

### Tissue motion-stabilized heart imaging window chamber system for IVM

**Figure 1:**
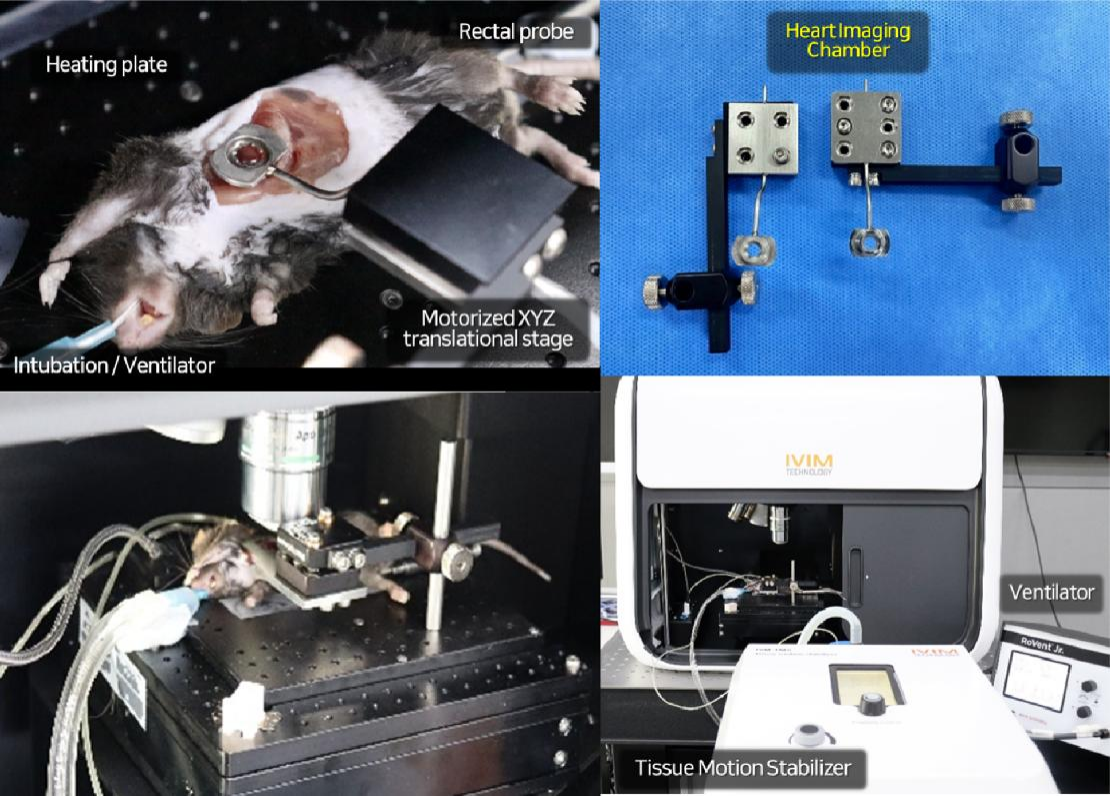
Suction-based motion-stabilized intravital heart imaging window chamber system. A heart-specialized imaging window chamber system for intravital cardiac tissue imaging, which is composed of a heart imaging window, vacuum-applied tissue motion stabilizer, ventilator, and thermostatic body maintenance system.

We established the heart-specialized imaging window chamber for intravital imaging utilizing the suction-based tissue motion-stabilized system for live heart imaging (*Figure 1*).

To expose the cardiac tissue while maintaining the physiological condition of the live mouse model, thoracotomy was performed during respiratory intubation with a mechanical ventilator and homeostatic body temperature regulation with a bidirectional thermostatic feedback system.

A heart imaging window chamber connected with a tissue motion-stabilized system was applied to the cardiac tissue after thoracotomy. Through the minimal level of negative pressure of vacuum (890–920 mbar) applied to the imaging window, making a closed chamber system in between the tissue and coverslip, cardiac tissue is attached to the coverslip and temporarily stabilized for stable visualization of cellular dynamics in the heart.

**Figure 2:**
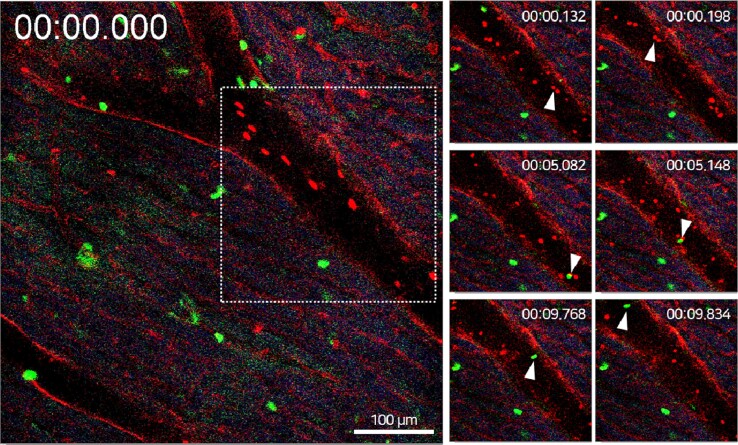
*In vivo* real-time cellular imaging of immune cell and RBC flowing dynamics in the heart. Ultra-fast real-time recording of flowing neutrophil and RBC dynamics in the arteriole of the left ventricle in live heart tissue. Sub-second video-rate cellular trafficking of flowing LysM^+^ neutrophil and DiD-labelled RBC dynamics in cardiac vessels in LysM-GFP mouse was indicated by arrowheads. Scale bar, 100 µm.

Utilizing the intravital heart imaging window system, we successfully obtained the real-time video-rate cellular dynamic recordings and image analysis of the cellular trafficking of circulating LysM + immune cells and flowing RBCs in the microvasculature of the left ventricle in the heart of the live mouse model (*Figure 2*). Adoptively transferred DiD-labelled erythrocyte dynamics were trafficked in sub-second frame rate (15 frames/s), which can finally derive the microvelocity of blood flow in the arterioles and venules in the cardiac tissue.

The rolling and circulating dynamics of cardiac tissue-resident neutrophils in the artery were captured sequentially. From the quantitative analysis of cell dynamics, the cellular velocity of adhering, crawling, and free-flowing neutrophils (mean velocity = 1.661 mm/s, *n* = 2) in the blood vessel is relatively slower than that of RBC flows (mean velocity = 9.221 mm/s, *n* = 3) in the heart of a normal mouse model. Additionally, the migration velocity of extravasated cardiac neutrophils within the cardiac tissue parenchyma in the steady state (mean velocity = 1.09 μm/s, *n* = 2) can be assessed to elucidate cellular immune responses in normal and diverse inflammatory states.

### Automatic motion compensation function to compensate cardiac muscle contraction for intravital heart imaging

To acquire the high-quality image sequences of highly contractile cardiac tissue *in vivo*, post-motion compensating process can be the great alternatives for intravital imaging as well as physically stabilized system. Although the cardiac muscle is well stabilized to the window chamber for intravital imaging, the innate contractile movement of the heart makes the motion-induced interruptions in the microregion of imaging interest.

**Figure 3:**
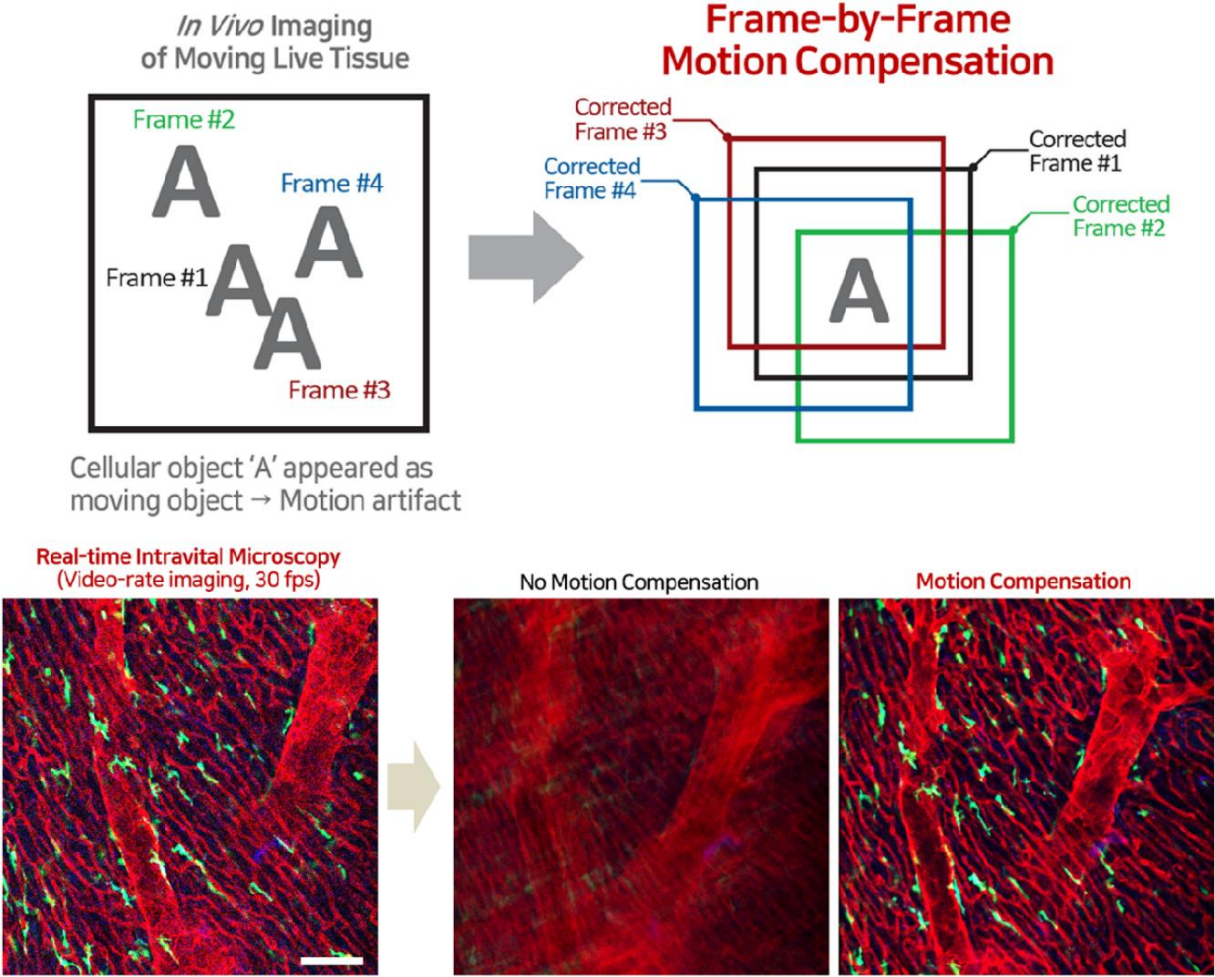
AI-based automatic motion compensation function for intravital heart imaging. Automatic motion correction proceeded for the real-time image frames based on the channel of the reference object ‘A’. The side-by-side comparison from *in vivo* heart images with microglial cells and cardiac vasculature; image from the typical imaging system without motion compensation and the final output image after motion correction. Scale bar, 100 µm.

AI-based post-processing motion compensation function highly minimized the live tissue-driven motion artefacts during real-time acquisition (*Figure 3*). Selective extraction and movement correction of the image frames sequentially acquired in real-time frame rate (∼30 frames/s) according to the reference object ‘A’, which is an unmoving landmark, derive fast automatic motion compensation during intravital microcardiac imaging of the beating heart.

In this study, blood vessel structure is the stationary landmark, whose colour channel (RFP) is set and recognized as a reference channel for compensating the cardiac motion artefact. During motion compensation in the 30-frame averaging setting, 30 frames collected over 1 s will be examined in real time. From the 30 frames acquired per second, frames with the red blood vessel structure at the same position as the initial frame are selectively extracted and averaged to produce a high-fidelity motion-compensated image output.

In the absence of motion compensation feature, image quality is compromised by blurring and shifting artefacts attributed to the tissue movement. However, through the integration of AI-based motion compensation, real-time acquisition of high-quality images with high spatiotemporal resolution facilitates precise quantitative analysis of cellular movement and dynamic measurements of fluorescence intensity.

### Intravital label-free SHG imaging of cardiac muscle with multiphoton microscopy

SHG imaging microscopy characterizes the establishing structures of muscles and fibrils in the cardiac tissue *in vivo*.^30,31^ For the label-free SHG imaging, multiphoton microscopy with non-linear pulsed laser visualizes the intrinsic signals from repetitive biostructures such as the muscle sarcomeres, myofibres, and fibrillar collagens.^30^

**Figure 4:**
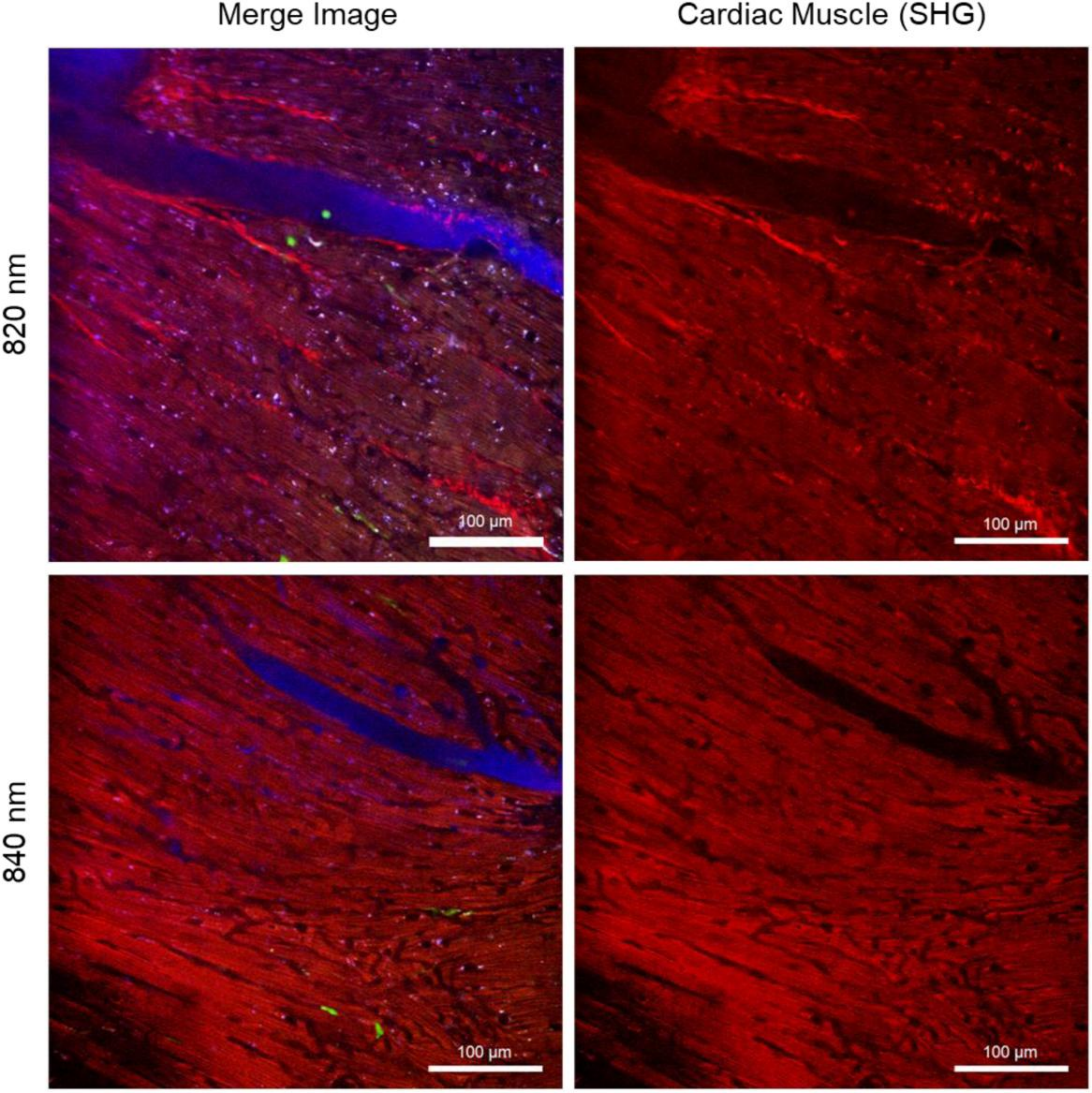
Label-free SHG imaging of cardiac muscle in the heart *in vivo*. SHG imaging of cardiac muscle structures including muscle sarcomeres, myofibrils, and collagen fibres, and two-photon fluorescence imaging of GFP^+^ neutrophils resident in cardiac tissue and vasculatures was identified in the heart in the live mouse model. Scale bar, 100 µm.

We identified the intravital cardiac muscle structures in the heart at 820–840 nm of excitation wavelength in two-photon microscopy utilizing heart motion-stabilized imaging window chamber. Simultaneous visualization of SHG striated muscles and GFP + neutrophils by SHG two-photon microscopy revealed the biodistributions and structural variations of sarcomeres and fibrillar organization in live heart (*Figure 4*). Dynamics immune cell features of slowly crawling LysM + neutrophils along the cardiac vessels and striated muscle-resident immune cells located in between of myofibrils were clearly identified *in vivo*.

## Discussion

IVM enables a dynamic 3D imaging of various cellular-level dynamics such as cell trafficking, cell–cell interaction, and cell–microenvironment interaction inside the living preclinical animal model *in vivo*. However, intravital cardiac microimaging has been quite challenging due to the high level of tissue movement and invasive surgical procedures.^32,33^

In this study, we established the optimized imaging protocols for intravital heart imaging in live mouse model utilizing tissue motion-stabilized heart imaging window chamber and AI-based motion compensation function. Moreover, in combination of two-photon non-linear optical microscopy, intrinsic label-free SHG imaging of cardiac muscles and multiphoton fluorescent imaging of neutrophils and vessels in the heart were clearly delineated *in vivo*.

Intravital heart microscopy imaging technology presented in this study demonstrates technical advances over previous methods in several key aspects including maintenance of the physiological state, imaging field of view, compatibility with other microscopy systems, and processing and acquisition time for motion compensation. Although continuous heart monitoring is limited to a maximum of 6 h due to the risk of tissue collapse and dryness resulting from prolonged thoracic openings, it has been shown that general physiological parameters of live mouse such as blood flow rate (8∼10 mm/s), body temperature (36∼37°C), and heart rate (550∼620 bpm) are within normal range and well maintained during imaging sessions. Nonetheless, alternative protocols utilizing endoscopy^26^ or suction-based implanted windows developed referring to previous implant technology^34^ should be employed for a minimally invasive thoracotomy approach to continuous intravital cardiac imaging.

The heart imaging chamber system is designed to enable the epicardium imaging through an 8 mm circular cover-glass window, facilitating simultaneous monitoring of coronary microvasculature such as cardiac arteries, veins, and capillaries, as well as muscle myofibres across various regions of cardiac tissue. In addition, it is applicable with conventional confocal and two-photon microscope integrating this chamber into any animal stage mount. Furthermore, this imaging technology has superiority in real-time processing and acquisition for motion compensation. A conventional motion correction technique relies on retrospective electrocardiogram (ECG) gating, extracting images selectively at specific point in respiratory and cardiac cycles, which necessitates the acquisition of images over a minimum of five consecutive cardiac and respiratory cycles, each taking at least 1 s to acquire a single motion-compensated image.^24,35^ However, AI-based post-processing strategy implements ultra-fast, high signal-to-noise motion correction at an acquisition speed of 10 fps without oversampling frame acquisition, facilitating the capture and analysis of rapid cell dynamics in the cardiac microenvironment. To further enhance dynamic analysis, post-processing motion correction is applied to real-time video recordings at 30 frames/s without time compensation, enabling more accurate measurement of ultra-fast biological dynamics. For more advanced imaging analysis demanding the highest spatiotemporal resolution, such as monitoring specific neuronal activity within the intact heart, the high-end algorithms including AI-based denoising functions for real-time video recordings must be further developed for real-time image acquisition with superior signal-to-noise ratios and increased temporal resolution. In addressing practical challenges, the rapid and sophisticated processing demands a high-performance computer with advanced specifications. For reference, it includes components such as an i7-9700K CPU running at 3.60 GHz with 8 cores operating at 3600 MHz, accompanied by 32 GB of RAM, a 500 GB SSD, and a GeForce RTC 2070 Super 8 GB graphics card.

The utilization of fluorescent reporter engineering technology enables intravital observation and evaluation of cellular and molecular dynamics in high signal-to-noise ratios, with minimal invasiveness at a high-throughput rate. This capability expedites the interpretation of cellular pathways such as cell communication, cell division, cell death, cell migration, and flow in diverse physiological conditions.^36,37^ In this study, aimed at elucidating the physiology of cardiovascular system including vascular networks and immune responses, LysM-GFP transgenic reporter mouse model,^28^ in which endogenous neutrophils, monocytes, and macrophages are labelled to express GFP, was utilized by transferring labelled RBC for *in vivo* blood and immune analysis. In steady-state condition, blood flow and leucocyte dynamics are assessed to be aligned with the result of previous report obtained with optical coherence tomography (OCT), confocal, and multiphoton fluorescence microscopy.^24,38^ IVM in cardiology provides insights into understanding comprehensive vascular immuno-pathophysiology underlying various cardiovascular disorders.

Intravital two-photon microscopy system with the optimized *in vivo* heart imaging window chamber system represents a promising approach for a long-term 3D fluorescence and label-free SHG intravital imaging of various cellular dynamics of tissue-resident immune cells, vessels, muscles, and circulating blood cells in the heart of live animal models. Intravital heart imaging protocol established in this study serves as a critical tool for preclinical investigations in cardiovascular research, allowing real-time visualization of diverse cellular activities implicated in cardiovascular pathologies. Specifically, it facilitates *in vivo* monitoring of microvascular blood circulation and acute inflammatory responses in condition of myocardial infarction, cardiac fibrosis in dystrophic muscles in Duchenne muscular dystrophy, and cardiac muscle contractility in atherosclerotic cardiovascular disease models. Furthermore, the versatility of this imaging protocol extends its application to other dynamically moving organs such as the lung, thymus, and uterus, further expanding research capabilities. This innovation in intravital cardiac microscopy is expected to significantly advance our comprehension and development of novel pharmaceutical therapeutics or preventive technologies for cardiovascular diseases.

## Data Availability

The data underlying this article will be shared on reasonable request to the corresponding author.

## Lead author biography

Dr Soyeon Ahn specializes in multimodal intravital imaging utilizing advanced window chamber systems in live mouse models. She has extensive *in vivo* research experience in comprehensive multiphase spatiotemporal visualization and imaging analysis of various organs, including the lymph node, lung, liver, spleen, and brain, using a video-rate intravital laser scanning microscopy system. As a research scientist at IVIM Technology, Dr Ahn continues to improve preclinical research performance by optimizing and expanding intravital imaging protocols and methods.
