# Correction to: Intravital imaging of cardiac tissue utilizing tissue-stabilized heart window chamber in live animal model

**DOI:** 10.1093/ehjimp/qyae095

**Published:** 2024-09-24

This is a correction to: Soyeon Ahn, Jung-yeon Yoon, Pilhan Kim, Intravital imaging of cardiac tissue utilizing tissue-stabilized heart window chamber in live animal model, *European Heart Journal - Imaging Methods and Practice*, Volume 2, Issue 1, January 2024, qyae062, https://doi.org/10.1093/ehjimp/qyae062

In the originally published version of this manuscript, an incorrect image was included in the Lead author biography section.

This has been corrected in the article.

